# Giant polypoid gastric heterotopia in the small intestine in a boy: A case report and literature review

**DOI:** 10.1097/MD.0000000000005854

**Published:** 2017-01-10

**Authors:** Jing Cai, Haibo Yu

**Affiliations:** Department of Digestive Diseases, Wenzhou Central Hospital, The Dingli Clinical Institute of Wenzhou Medical University, Wenzhou, Zhejiang, P.R. China.

**Keywords:** anemia, endoscopy, heterotopic gastric mucosa

## Abstract

**Rationale:**

Heterotopic gastric mucosa has been described at various locations of the body; however, the polyp composed of heterotopic gastric mucosa in the small intestine is rare.

**Patient concerns:**

A 15-year-old boy visited us for investigation of recurrent episodes of melena. Capsule endoscopy (CE) revealed a polypoid tumor in the ileum, with an active nearby hemorrhage. Contrast-enhanced computed tomography (CECT) showed a tumor in the right quadrant of the abdomen, with a diameter of about 18 × 14 mm.

**Diagnoses:**

The patient was diagnosed with polypoid gastric heterotopia.

**Interventions:**

We performed an operation to resect the lesion.

**Outcomes:**

The patient recovered smoothly after surgery and was discharged on postoperative day 7 and followed up for 3 months. He has not experienced gastrointestinal intestinal (GI) symptoms up to now.

**Lessons:**

Giant polypoid gastric heterotopia in the small intestine is extremely rare, which can express as an occasional finding with or without symptoms. Surgical resection is the preferred therapy when symptoms appear.

## 1 Introduction

Heterotopic gastric mucosa (HGM) has been described at various locations of the body, including all levels of the gastrointestinal tract.^[1]^ It causes problems such as intestinal obstruction, intestinal bleeding, volvulus, perforation, and even death.^[2–5]^ However, giant polypoid gastric heterotopia in small intestine is rare findings. Here, we reported a case of a boy who was diagnosed with a giant polyp in ileum composed of heterotopic gastric mucosa. To our knowledge, this is the first case that reveals a giant polypoid gastric heterotopia in the small intestine.

## 2 Case presentation

**Figure 1:**
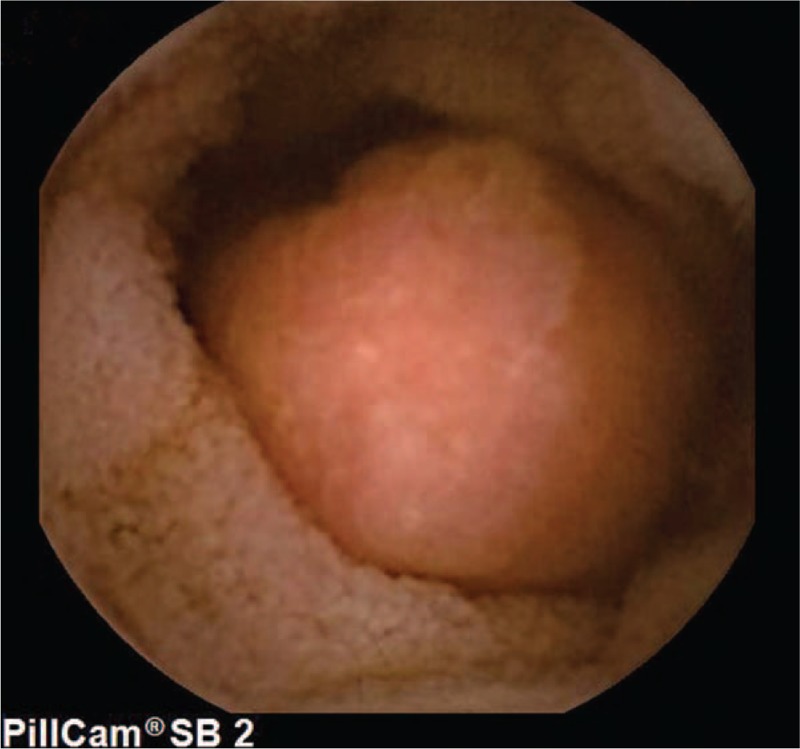
Capsule endoscopy (CE) demonstrated a polypoid tumor of about 2 cm in diameter in the ileum, with an active nearby hemorrhage. CE = capsule endoscopy.

**Figure 2:**
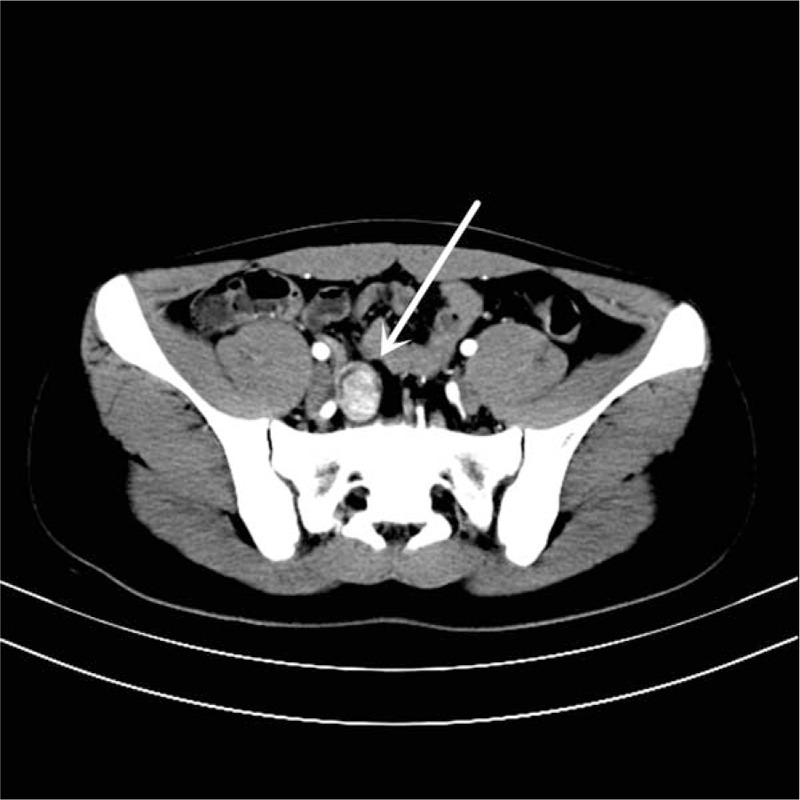
CECT demonstrated a tumor in the right quadrant of the abdomen, size of about 18 × 14 mm in diameter (arrow). CECT = contrast-enhanced computed tomography.

**Figure 3:**
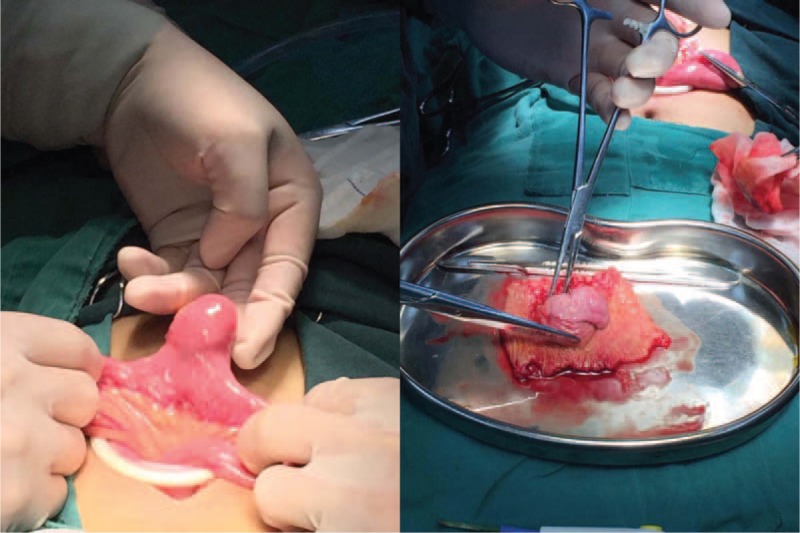
The resection specimen was a polyp (2.2 × 1.5 × 1.2 cm).

**Figure 4:**
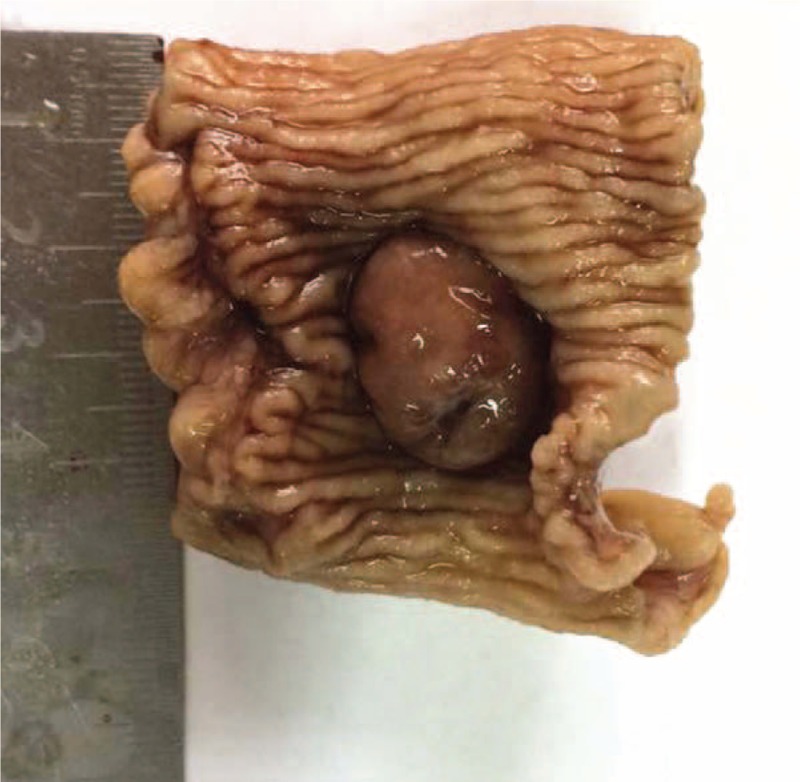
Macroscopically, most of the mucosa in the polyp was similar to the mucosa of the stomach.

**Figure 5:**
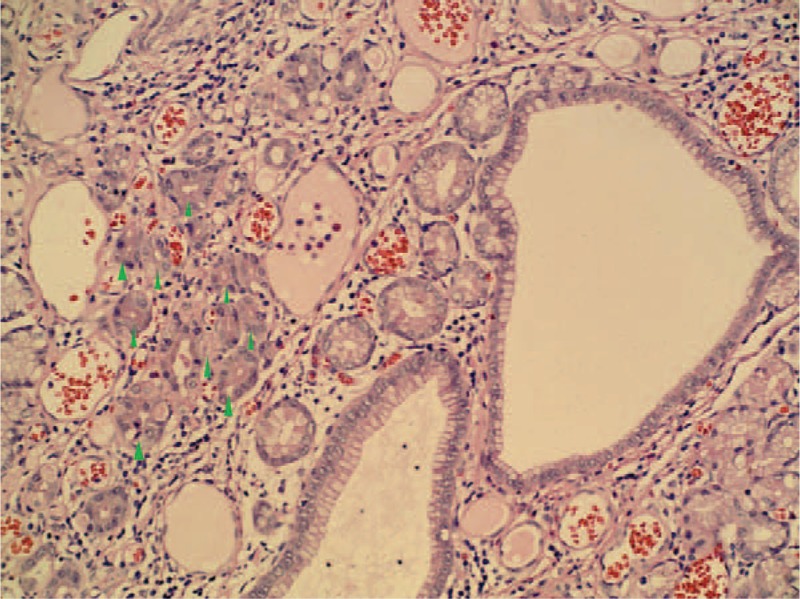
Histological examination revealed that the polyp contained heterotopic gastric mucosa with well-developed gastric gland (magnification × 200 arrow).

**Figure 6:**
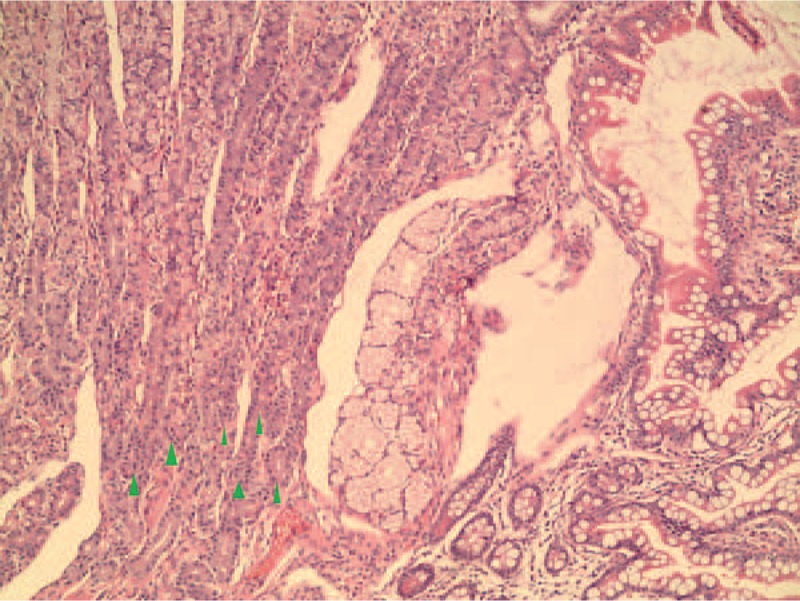
This section of a polyp shows various gastric glands (magnification × 100 arrow).

A 15-year-old boy visited us for investigation of intermittent episodes of melena for a week. The patient had no history of alimentary tract hemorrhage, infection, trauma, or medication history. Hematological examination showed a hemoglobin (Hb) value of 7.5 g/dL. Esophagogastroduodenoscopy (EGD) and colonoscopy revealed negative result. Capsule endoscopy (CE) demonstrated a polypoid tumor of about 2 cm in diameter in the ileum, with an active nearby hemorrhage (Fig. 1). However, CECT demonstrated a right lower-abdominal tumor, with a size of about 18 × 14 mm in diameter (Fig. 2). There was heterogeneous enhancement during the arterial phase, and the density was high in portal and delayed phases. An equivocal diagnosis was considered based on the findings with the CECT, which suggested a gastrointestinal stromal tumor (GIST), hemangioma, or adenoma. Laparotomy was performed under general anesthesia. We resected the intestine with lesion, and an end-to-end anastomosis of the small intestine was performed. The specimen was a sessile polyp (22 × 15 × 12 mm) (Fig. 3). Macroscopically, most of the mucosa in the polyp was similar to the mucosa of the stomach (Fig. 4). Histological examination revealed that the polyp contained heterotopic gastric mucosa with the well-developed gastric gland (Figs. 5 and 6). The patient was diagnosed with polypoid gastric heterotopia. The patient recovered smoothly after surgery and was discharged on postoperative day 7 and followed up for 3 months. He has not experienced gastrointestinal intestinal (GI) symptoms up to now.

## 3 Discussion

HGM of the small intestine is a congenital disorder with a variable clinical presentation. It may occur in the duodenum, esophagus, colon, and most commonly occurs in ileum in Meckel's diverticulum (MD) and gastrointestinal duplications.^[6,7]^ Poindecker reported the first case of HGM in 1912.^[8]^ Subsequently, many cases of small intestinal HGM have been reported. The incidence of HGM in the esophagus varies widely from 0.1% to 13.8%, and that of HGM in duodenal varies from 0.5% to 8.9%.^[9]^ HGM usually presents as an “inlet patch” in the proximal esophagus, as polypoid masses in rectum, or as nodular tumors in the duodenum.^[10,11]^ Polypoid lesions composed of gastric mucosa in the small intestine are rare. The vast majority of reported cases occur as small metaplastic nodules or polyps in duodenum in association with other disease processes.^[12,13]^

HGM can cause intestinal perforation, gastrointestinal bleeding, and intestinal obstruction.^[14–18]^ It also can cause failure to thrive because of the chronic abdominal pain associated with recurrent episodes of vomiting and diarrhea.^[19]^ Our case was unique in that the lesion appeared as a giant polyp composed of heterotopic gastric mucosa in the small intestine. Polypoid gastric heterotopia is normally present sporadic, small in dimension and with no symptoms. The clinical feature depends on the size of the polypoid gastric heterotopia. It can cause intestinal and airway obstruction.^[20,21]^ However, giant polypoid gastric heterotopia in small intestine caused gastrointestinal bleeding has not been reported yet. The natural history of polypoid gastric heterotopia is deficient in clearness and currently there are no available guidelines for surveillance or treatment. The mechanism for the development of polypoid gastric heterotopia in the small intestine remains to be seen. There are various theories that are used to explain the etiology. (1) It occurs between the fourth and seventh week of fetal development due to an erroneous differentiation of the pluripotent stem cells of the endodermis. (2) It occurs due to a failure in the descent of the proximal intestine. (3) It is the result of an anomalous regeneration under inflammatory conditions, such as gastric metaplasia in Barrett's esophagus.^[22,23]^ 4) It occurs due to Cdx2 modify the expression of molecules. Cdx2 stimulates markers of enterocyte differentiation.^[24]^ Null mutation of Cdx2 lead to the development of ectopic lesions with a gastric phenotype in the midgut endoderm.^[25]^ The study present that mice in which Cdx2 gene had been inactivated developed multiple intestinal polyp-like lesions in the mouse esophagus and stomach.^[26]^

The clinical diagnosis of polypoid gastric heterotopia may be difficult before surgery due to its rarity and urgent presentation. It is often discovered during laparotomy and confirmed upon pathologic examination post-operatively. There are no endoscopically distinctive features of polypoid gastric heterotopia in the intestine. It is difficult to differentiate polypoid gastric heterotopia in the intestine from other intestinal diseases, such as GIST, hemangioma, adenoma, or inversion of intestinal diverticulum. History-taking and physical examination are required, which help to suspect the potential diseases involved. Cross-sectional imaging such as CECT should be done as well, followed by the latest enteroscopy, capsule endoscopy, and deep enteroscopy according to the patient's conditions. CE is a viable and safe option.^[27]^ Tc-99m pertechnetate scan may be useful depending upon the location and size of the heterotopic tissue.^[28]^ The final diagnosis depends on the histological results from surgical specimens.

## 4 Conclusions

We are presenting a rare case with giant polypoid gastric heterotopia in the ileum. We should pay attention to diagnosis of polypoid gastric heterotopia. Endoscopy combined with histopathologic examination is definitely a mandatory method in clinical diagnosis. Surgical resection is the preferred therapy when symptoms appear.
